# An observational study of fixed-dose *Tanacetum parthenium* nutraceutical preparation for prophylaxis of pediatric headache

**DOI:** 10.1186/s13052-019-0624-z

**Published:** 2019-03-12

**Authors:** Filomena Moscano, Michela Guiducci, Lucia Maltoni, Pasquale Striano, Maria Giuseppina Ledda, Francesco Zoroddu, Umberto Raucci, Maria Pia Villa, Pasquale Parisi

**Affiliations:** 1 Child & Adolescent Neuropsychiatry, Hospital S. Orsola Malpighi, Bologna University, Bologna, Italy; 2 Child Neurology, Pediatric Headache & Sleep Disorders Centre, Chair of Pediatrics, NESMOS Department, Faculty of Medicine and Psychology, Sapienza University, Via Di Grottarossa, 1035–1039, 00189 Rome, Italy; 3 Paediatric Neurology and Muscular Diseases Unit, Department of Neurosciences, Rehabilitation, Ophthalmology, Genetics, Maternal and Child Health, University of Genoa, ‘G. Gaslini’ Institute, Genoa, Italy; 4 Child & Adolescent Neuropsychiatry Unit, “Antonio Cao” Paediatric Hospital, “G.Brotzu” Hospital, Cagliari, Italy; 5 Pediatric Headache Center, Neurology Unit Hospital, University of Sassari, Sassari, Italy; 6 Pediatric Emergency Department, Bambino Gesù Children’s Hospital, IRCCS, Rome, Italy

**Keywords:** Nutraceuticals, Pediatric migraine, Observational study, *Tanacetum parthenium*, Prophylaxis

## Abstract

**Background:**

Migraine is one of the most prevalent chronic pain manifestations of childhood. Despite the multitude of available treatments, parents are often concerned about chronic therapies and pediatricians have insufficient confidence in prescribing prophylactic drugs. Therefore, there is now growing interest for natural supplements used to control recurrent migraine headaches. Such approach may increase acceptance and adherence to long-term prophylaxis therapy in children.

**Methods:**

This is an observational multicenter study performed in children (*n* = 91) with migraine, with (MO) or without aura (MA), or tension-type headache (TTH). A fixed-dose *Andrographis paniculata*, CoQ10, riboflavin, and magnesium, was administered for 16 weeks. Patients were evaluated at baseline (T0), at week 8 (T1) and at the end of treatment at week 16 (T2). A follow-up period occurred at week 20 (T3) and week 32 (T4).

**Results:**

The herbal supplement significantly reduced the frequency of headaches in TTH patients during treatment period (T0: 11.97 + 1.92 vs T2: 5.13 + 1.93; *p* < 0.001) and the efficacy was maintained after 16 weeks of treatment withdrawal (T4: 4.46 + 1.75; *p* < 0.001 vs T0). The frequency of migraine attacks was also reduced in the MO group during treatment (T0: 9.70 + 0.96 vs T2: 4.03 + 0.75; *p* < 0.01) and after withdrawal (T4: 2.96 + 0.65; p < 0.01 vs T0). Conversely, MA patients showed reduction in migraine’s frequency during treatment (T0: 8.74 + 1.91 vs T2: 3.78 + 2.02; p < 0.01) but not at the end of the study (T4: 5.57 + 3.31; *p* > 0.05 vs T0).

TTH patients did not report significant improvement of pain intensity. A significant effect was observed in the MO group during treatment (T0: 3.06 + 0.11 vs T2: 2.14 + 0.19; *p* < 0.001) and after treatment withdrawal (T4: 2.20 + 0.21; p < 0.001 vs T0). Likewise, MA group showed a significant treatment effect (T0: 2.57 + 0.20 vs T2: 0.86 + 0.45; p < 0.001) and the efficacy persisted at the end of the study (T4: 1.00 + 0.58; p < 0.001 vs T0).

**Conclusion:**

This fixed-dose *Tanacetum parthenium* preparation improved headache frequency and pain intensity in children affected by TTH. Despite the main limits, this study supports the use of nutraceutical in pediatric headache/migraine.

## Background

Migraine is one of the most prevalent neurological symptom and chronic pain manifestation of childhood, affecting up to 10% of children between the ages of 5 and 15 years and up to 28% of adolescents aged from 15 to 19 years [1]. Migraine is defined as an episodic pain disorder that can vary from an occasional occurrence to a daily frequency and can subsequently become a very disabling disorder, with a substantial effect on the child’s quality of life [2, 3] . The disease can cause a significant distress as for the child, as for the entire family. If not successfully treated, frequent and recurrent headaches can impact families with significant disability due to loss of school, work, and social activities. As for adults, migraine treatment traditionally includes acute therapy for aborting migraine attacks and prophylactic treatment for reducing the frequency, duration and severity of attacks [4]. Notably, headache amongst children has a high risk of development into a chronic condition and persisting into adulthood [5]. Thus, in children an effective early treatment would be expected to impact disease progression before many of the refractory aspects seen in adults become established [6]. There are several classes of medications that may be used for prophylactic therapy like antidepressants, anticonvulsants, antihistamines, beta-adrenergic receptor blockers, and calcium ion channel antagonists, botulinum toxin [7, 8]. Due to the potential limitations of conventional treatment of obtaining a satisfactory response to pain in many cases of primary headache, various alternative treatments are sought by patients; in particular, parents ask healthcare professionals to provide migraine relief for their children. Moreover, even clinicians prefer to avoid prescription of prophylactic therapies in children due to the poor evidence of efficacy and significant potential adverse effects in this population [9] and the well-known “placebo effect” observed in children [10].

There is now growing interest for natural supplements for migraine and headache treatment. Due to the low side effects, several studies support the efficacy of natural treatments, in particular nutraceuticals, in the prophylactic treatment of migraine in children and adolescents, even if the evidences are still limited [11].

Nutraceutical is a term derived from “nutrition” and “pharmaceutical”, indicating “food, or parts of a food, that provide medical or health benefits, including the prevention and treatment of diseases”. Nutraceutical treatment consists of taking vitamins, supplements (like magnesium, riboflavin, coenzyme Q10, and alpha lipoic acid) and herbal preparations (like feverfew and butterbur). This type of approach enhances patient acceptance and adherence to long-term prophylaxis therapy combined with appropriate information on treatments’ efficacy and safety [11].

Magnesium (Mg^2+^) has a role in various biological processess and it is involved in ATP production and function and in the control of vascular tone and it binds to NMDA receptors; its role has been suggested because of magnesium deficiency demonstrated in headache physiopathogenesis [11]. Coenzyme Q10 (CQ10) has shown a crucial role in sustaining mitochondrial energy stores, as a carrier in the mitochondrial electrons transport chain; furthermore it has antioxidant proprieties. A role of Cq10 in migraine pathogenesis has been postulated because migraineurs have difficulties in energy production [11]. Riboflavin (B2 vit) has two active coenzyme forms cofactors in oxidation–reduction reaction of flavoproteins and seems to reduce symptoms in patients with mitochondrial dysfunction [11]. The anti-migraine activity of Tenacetum Partheniunim (Feverfew) is probably related to the inhibition of oxide nitric synthesis, to the cytokines induction, to the release of serotonin from the platelets and to the inhibition of the Calcitonin Gene-Related Peptide (CGRP) release from the trigeminovascular system [11].

To this aim, keeping in mind all the above reported considerations, we have performed a multicenter, prospective study to evaluate the efficacy and safety of Partena® tablets (a combination of Mg^2+^ 169 mg, CoQ10 20 mg, VitB2 4,8 mg, Feverfew 150 mg-1,2 mg Parthenolides and *Andrographis paniculata* 100 mg) available in Italy as a dietary supplement (Italian Registry of Supplements code 64289) in a population suffering from migraine/headache in the developmental age, referring to headache outpatient centers.

## Methods

### Study design

This was a multicenter, prospective, observational study in children and adolescents with migraine (with or without aura) or tension-type headache. From January 2016 to December 2017, patients from 9 to 18 years were consecutively enrolled from five different neurologic pediatric centers in Italy (Sapienza University-Sant’Andrea Hospital, Rome, Gaslini Childrens’ Hospital, Genoa, Sant’Orsola-Malpighi Hospital, Bologna, “Businco Hospital”, Cagliari, and University Hospital, Sassari). The study was conducted in a context of routine practice without any additional or unusual procedure of diagnosis or surveillance. The study was also conducted according to the Declaration of Helsinki. Written informed consent from a parent or guardian and, when appropriate, child assent were obtained prior to study partecipation. Patients underwent a baseline evaluation performed by Pediatricians or Child Neurologists with expertise in pediatric headache, in order to confirm the diagnosis and their eligibility for the study. During the assessment patients reported their age, medical history, migraine family history, headache type and localization. Inclusion criteria were migraine with aura (MA), without aura (MO) or tension-type headache (TTH), as defined according to the International Classification of Headache Disorders, 3rd Edition [12]. Moreover, a headache frequency of 4 or more days/month should have been reported on a headache diary over a pre-baseline period, for at least the latest 3 months. Patients were excluded if they previously used headache prophylactic treatment (drugs, nutritional supplement or psychotherapy); all secondary headache disorders were also excluded.

Partena® tablets, 1 tablet, twice a day, at regular interval, was administered by Parents’ children, for the first 4 weeks, following by 12-weeks constant-dose phase of 1 tablet per day. Adherence and compliance to therapy and side effects were recorded in the clinical headache diary, during all phases of study. Patients were evaluated at baseline (T0) at week 8 (T1) and at the end of treatment at week 16 (T2). At week 20 (T3) and week 32 (T4), each enrolled patient was re-evaluated *(follow-up period).* Obviously, before entering the baseline phase, children had to meet inclusion and exclusion criteria, as specified above.

### Pain assessment

Patients completed a daily headache diary, in accordance with the NINDS Common Data Elements [13]. A headache day was defined as any day during which a headache occurred within a 24-h period, starting at midnight. The four-point pain scale (none, mild, moderate, severe) has been used in this study to assess treatment efficacy.

### Safety

Safety was assessed with the use of adverse-event reports that were collected from parents and patients by means of a structured interview.

### Statistical analysis

A paired analysis of variance (ANOVA) and post-hoc paired t-test analysis was performed to analyze comparison during treatment and follow-up period. Data were given as mean ± standard deviation (SD). The two-sided Fisher exact test was performed for contingency analysis. Patients who did not maintain compliance to treatment of at least 85% till the end of the study were considered as censored data for the “pre-protocol” analysis. We accepted as significant *p* values of less than 0.05.

## Results

A total of 91 patients **(mean age 14.4** **+** **2.2; males 34% and females 66%)** were enrolled (demographic and clinical variables at baseline are shown in Table 1.). Treatment with Partena was substantially well tolerated, but 4.4% of patients interrupted the treatment due to gastrointestinal symptoms (nausea and diarrhea). A small group of patients (8.8%) interrupted the study protocol due to treatment inefficacy while the 7.7% of patients was lost at follow-up.

**Table 1:** Demographic and clinical characteristics of patients at baseline (*n* = 91)

Variable	mean (± s.d.) *or* n (%)
Age, years	14,4 ± 2,2
Female	60 (66%)
Male	31 (34%)
Tension-type Headache (TTH)	20 (22%)
Migraine with aura (MA)	7 (8%)
Migraine without aura (MO)	64 (70%)

In the intention-to-treat analysis (*n* = 91) the percentage of patients showing a significant reduction of 50% or more in the number of headache episodes at the end of the 16-weeks treatment period (T2) was 50% in the TTH group, 55% in the MO group and 85% in the MA group. A significant maintenance of this effect after 16 weeks from the treatment withdrawal (T4), was seen for TTH (50%) and MO group (53%), but not for MA patients which showed a slight but significant reduction in the efficacy respect to the treatment period (71%, *p* < 0.01 respect to T2).

The percentage of patients who completed the study was 75% of TTH, 83% of MO group and 100% of MA.

**Fig. 1:**
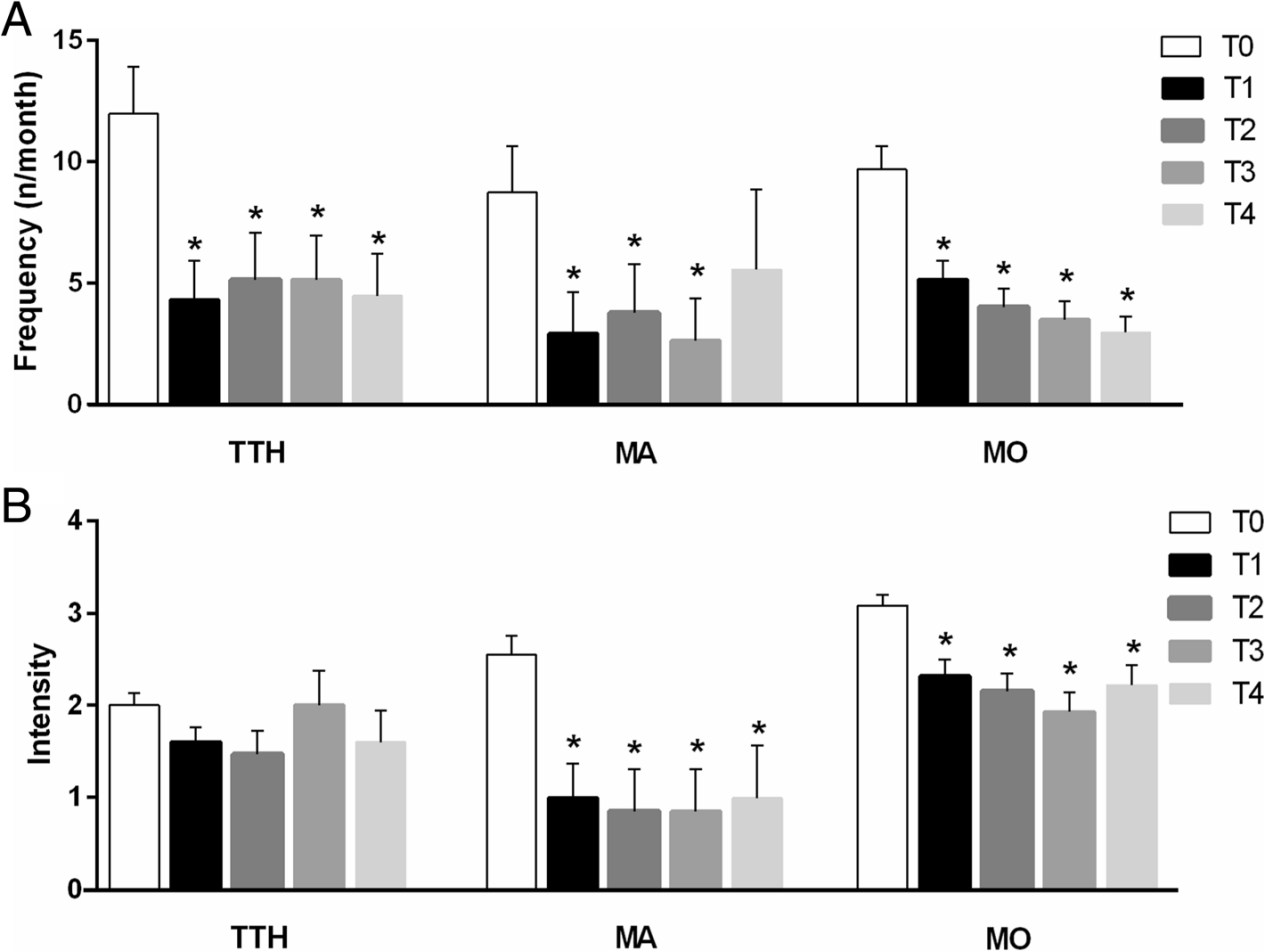
Results for frequency of attacks (**a**) and Intensity of pain (**b**) at baseline and at the post-baseline time points by subgroup of diagnosis (values are means). Migraine with (MO) or without aura (MA), tension-type headache (TTH). Bars are standard deviations. *p < 0.05

In the analysis per-protocol (*n* = 72), Partena significantly reduced the frequency of headaches respect to baseline in TTH patients (F(1.574, 22.04); *p* = 0.004) during the period of treatment assumption (T0: 11.97 + 1.92 vs T2: 5.13 + 1.93; *p* < 0.001). The efficacy was maintained even after 16 weeks of treatment withdrawal (T4: 4.46 + 1.75; p < 0.001 respect to T0). A significant reduction of migraine frequency was also found for MO group (F(2.740, 131.5); *p* < 0.0001) both during treatment (T0: 9.70 + 0.96 vs T2: 4.03 + 0.75; *p* < 0.01) and after 16 weeks of treatment withdrawal (T4: 2.96 + 0.65; *p* < 0.01 respect to T0). Conversely, MA patients showed a significant effect in reducing headache frequency during treatment (T0: 8.74 + 1.91 vs T2: 3.78 + 2.02; *p* < 0.01) but not until the end of the study (T4: 5.57 + 3.31; *p* > 0.05 respect to T0). (Fig. 1a).

In the assessment of pain intensity, TTH patients did not show any significant amelioration (F (2.463, 34.49); *p* > 0.05). Interestingly, a significant effect of treatment was found in MO group (F(3.471, 166.6); p < 0.0001). In particular, a reduction of pain intensity was found since 8 weeks of treatment (T0: 3.06 + 0.11 vs T1: 2.31 + 0.17; *p* < 0.001), remain significant at 16 weeks (T2: 2.14 + 0.19; *p* < 0.001 respect to T0) and at follow-up visits after treatment withdrawal (T4: 2.20 + 0.21; *p* < 0.001 respect to T0). Similarly MA group showed a significant treatment effect (F(1.459, 8.752); p < 0.01) with a good response since 8 weeks of treatment (T0: 2.57 + 0.20 vs T1: 1.00 + 0.38; p < 0.001), after 16 weeks (T2: 0.86 + 0.45; *p* < 0.001 respect to T0) and the efficacy was maintained until the end of the study (T4: 1.00 + 0.58; *p* < 0.001 respect to T0) (Fig. 1b). Globally, the reduction of pain intensity was higher, although not significantly, among MA respect to MO patients when evaluated during treatment administration (T2: 69% vs 29%; *p* = 0.12) and at the end of the study (T4: 67% vs 23%; *p* = 0.11).

## Discussion

Our study showed that administration of Partena® for a period of 16 treatment weeks reduced headache frequency in a sample of children and adolescence affected by TTH and Migraine. Moreover, in our observational study, treatment with Partena was associated with lower intensity of pain for migraine episodes among pediatric patients suffering from MA and MO. We used a combination of magnesium, riboflavin, CQ10, *Tanacetum parthenium*, molecules all arising in the guidelines of the American Headache Society (AHS) and American Academy of Neurology (AAN) [14] in addition to the *Andrographis paniculata* (plant extract that has shown analgesic and anti-edema activity in preclinical studies along with anti-nociceptive effects in an animal model of sensory hypersensitivity associated with migraine) [15]. Guidelines of the *Società Italiana Studio Cefalee* (SISC) support the use of feverfew, Mg^2+^, riboflavin and CQ10 a Level III recommendation [16].

*Tanacetum parthenium* (feverfew) has been used for centuries to treat pain and headache [17]. Several preclinical studies have identified different targets for parthenolide like inhibition of platelet aggregation, inhibition of serotonin release from platelets and white blood cells, reduction of iNOS (inducible nitric oxide synthase) activation, inhibition of nuclear factor-kappaB (a transcriptional factor involved in the mediation of pain and inflammation) and binding to and inhibiting IκB kinase complex (IKK) β that plays an important role in proinflammatory cytokine-mediated signaling [18, 19]. More recent pharmacological data revealed that antimigraine effect of parthenolide may derives from its ability to target transient receptor potential ankyrin 1 (TRPA1), inducing nociceptor desensitization, ultimately resulting in inhibition of Calcitonin gene-related peptide (CGRP) release within the trigeminovascular system [20]. Clinical evidences about the treatment of migraine are still controversial although the contradictory results may be attributed to the variations in the concentration and to the differences in the stability of the parthenolides. The most recent randomized controlled trial, using a stable feverfew extract, added some positive evidences about the efficacy of parthenium in the prophylactic treatment of migraine [17, 21]. Studies on Feverfew efficacy in children and adolescents with migraine are lacking [11].

The other active principles of Partena® currently available in Europe and USA as dietary supplements, have several epidemiologic, preclinical and clinical evidences supporting their usefulness in prophylactic treatment of migraine [21]. Several studies revealed decreased levels of the micronutrients riboflavin (Vit B2), magnesium (Mg) and CQ10 in plasma and brain of migraine patients [22]. On the other hand, numerous studies suggest a mitochondrial energy depletion in patients with migraine, and since all these molecules play an important role in the production of energy at mitochondrial level, multiple trials have assessed their efficacy in migraine prevention [23–25]. Riboflavin is a cofactor in oxidation-reduction reaction in the citric acid cycle and the electron transport chain, as such it plays a key role in the mitochondrial production of energy [21]. To date, evidence in headache treatment for pediatric age gave discordant results [9, 10, 26]. CQ10 is a vitamin-like compound needed for all cellular processes requiring energy. It is an electron-carrier, transferring electrons from complex I/complex II to cytochrome C in the mitochondrial electron transport chain [27]. Preliminary evidences appear to support efficacy for CQ10 in preventing migraines in children and adolescents, but remain insufficient to make a strong conclusion [26]. Mg^2+^ is necessary as a co-factor for proper functioning of the ATP-synthase. Furthermore, Mg^2+^ is the physiological antagonist at the NMDA-channel which is involved in the regulation of neuronal excitability and inhibition of cortical spreading depression (CSD) through several mechanisms involving serotonin receptors, NO synthesis/release as well as NMDA receptors [28]. The role of magnesium in the prophylactic treatment of pediatric migraine is still unclear and high-quality adequately powered trials are needed [26]. Andrographolide has a broad range of pharmacological properties, mainly an anti-inflammatory effect, by interfering with the production of inflammatory mediators, in particular cyclooxygenase (COX) enzymes and pro-inflammatory cytokines through the modulation of the NF-κB signaling network and the NO/iNOS pathway [29].

Nevertheless, it is conceivable to suppose that administration of key micronutrients and phytoextracts able to inhibit prostaglandin production and to interfere with the NO/iNOS pathway as well as mitochondrial production of energy, might prevent and/or reduce the number and intensity of the attacks. This is more interesting when considering that treating headaches in pediatric population is a continuous challenge for clinicians. Although migraine in children is phenotypically similar to adult migraine, medications efficacious in adults did not show similar efficacy in pediatric controlled clinical trial and none are currently FDA-approved for this age group [30, 31]. Therefore, it may be necessary to use medications off label strictly weighing up the benefits and risks.

This study was performed on a small sample and the clinical effects of nutraceutical have not been compared with placebo. In fact, a limitation of our study is linked to the absence of a control group and on short time of observation.

## Conclusions

To date there are scarce and contrasting evidences in favor of the most utilized drugs for prophylactic therapy in pediatric migraine and there are no significant differences between the high placebo response rate and the low drug response rate in pediatric age [9, 10]. Thus, the adult model of headache treatment, in which, for example, amitriptyline and topiramate, have been effective, may not apply to pediatric patients. In addition, the reported serious adverse events in children, do not show a favorable risk–benefit profile for the use of these therapies in pediatric migraine prevention [9].

On the other hand, our result clearly indicates that Partena® was able to reduce headaches frequency and pain intensity in children and adolescences suffering from migraine and TTH, therefore should be considered as a potential valuable prophylactic option for pediatric migraine. Although this study remains mainly observational, clear indications on the potential effectiveness, along with a favorable tolerability profile, of this combination on pediatric headache/migraine arose, and suggestion to perform further studies emerges to confirm the trend observed here. Randomized controlled trials are needed to further assess the effectiveness in nutraceuticals’ use for migraine treatment in child and adolescent.

## Abbreviations

AAN: American Academy of Neurology
AHS: American Headache Society
CI: Confidence Interval
ED: Emergency Department
FDA: food and drugs administration
IKK: inhibiting kinase complex
iNOS: inducible nitric oxide synthase
MA: Migraine Without Aura
MO: Migraine With Aura
NMDA: N-Methyl D-Aspartate
NO: nitric oxidase
OR: Odd Ratio
SD: Standard Deviation
SISC: Società Italiana per lo Studio delle Cefalee
TRPA1: transient receptor potential ankyrin 1
TTH: Tension-Type Headache
